# External quality assessment program for biochemical assays of human seminal plasma: a French 6-years experience

**DOI:** 10.1186/s12610-020-00116-2

**Published:** 2020-11-17

**Authors:** Safouane M. Hamdi, Erick Sanchez, Delphine Garimbay, Stéphanie Albarede

**Affiliations:** 1 Groupe de Recherche en Fertilité Humaine (Human Fertility Research Group), EA3694, Université Toulouse III - Paul Sabatier, Toulouse, France; 2 Laboratoire de Biochimie et d’Hormonologie, Institut Fédératif de Biologie, Hôpital Purpan, CHU Toulouse, 330, avenue de Grande-Bretagne TSA40031, 31059 Toulouse cedex, France; 3 CTCB, Centre Toulousain pour le Contrôle Qualité en Biologie Clinique, 33, Route de Bayonne, 31300 Toulouse, France

**Keywords:** Laboratory proficiency testing, ISO 15189, Seminal plasma, Biomarkers, Male genitalia, Citrate, Fructose, Glucosidase, Carnitine, Glycerophosphocholine

## Abstract

**Background:**

In 1999, despite a longstanding use, the WHO manual for the examination of human semen finally proposed to assay several biochemical components of the seminal plasma for a functional exploration of the male accessory glands. At the same time, an international effort was made to standardize laboratory tests and to increase their performance through ISO 15189 accreditation. In this setting, participation to relevant external quality assessment (EQA) schemes is an essential requirement for laboratories. To fulfil this injunction, we have organized an EQA program for seminal biochemistry using presumed commutable samples. In this study, we aimed to report an overview of the French laboratory offer, the kinds of assays used, their performance as well as their likelihood of satisfying ISO15189 requirements for EQA.

**Results:**

Between 2014 and 2019, we performed seven surveys. A median of six laboratories participated to each survey giving a ratio of one laboratory per 11.2 million inhabitants. Seven biomarkers are routinely assayed but the core set shared by all laboratories comprised citrate and zinc (prostate), fructose (seminal vesicles) and α-1, 4 glucosidase (epididymis). The use of CE-IVD marked methods concerned between 0 to 75% of overall assays. According to analytical specifications, 100% of laboratories results were compliant for zinc, 75% for citrate and α-1,4 glucosidase and 67% for fructose. By combining overall data in an empirical scoring system, we identified several types of seminal biomarkers: citrate, fructose and zinc appear as good candidates for a full accreditation, α-1,4 glucosidase still presents an analytical weakness, but prostatic acid phosphatase, free L-carnitine and glycerophosphocholine cannot be accredited in the current state.

**Conclusions:**

We organized the first French EQA program for seminal biochemistry to help local laboratories to face their legal requirement to be fully accredited by 2020. It could be improved still further but it gave us an oversight on the analytic landscape. Effective methods are available for a confident biochemical exploration of prostate and seminal vesicles. However, that of epididymis appeared unexpectedly fragile. This andrological issue should be addressed by dedicated recommendations from health authorities and scientific societies.

## Résumé

**CONTEXTE:**

Malgré un usage ancien, ce n’est qu’en 1999 que l’OMS propose dans son manuel d’examen du sperme humain de doser plusieurs composés biochimiques du plasma séminal pour l’exploration fonctionnelles des glandes annexes. A la même période, un effort international s’organise pour standardiser les examens de laboratoires et augmenter leur performance avec l’accréditation selon la norme ISO 15189. Dans ce contexte, la participation des laboratoires aux programmes d’évaluation externe de la qualité (EEQ) est une exigence fondamentale. Afin de les aider à faire face à cette obligation, nous avons organisé un EEQ pour la biochimie séminale basé sur des échantillons commutables. Dans ce travail, nous souhaitons rapporter une vue globale sur l’offre analytique française, sur les méthodes de dosage utilisées et leurs performances. Nous évaluons également leur potentiel à répondre aux exigences de la norme concernant l’accréditation.

**RESULTATS:**

Nous avons proposé entre 2014 et 2019 sept enquêtes. Une médiane de six laboratoires a répondu à chaque enquête, soit un ratio d’un laboratoire pour 11.2 millions d’habitants. Sept marqueurs séminaux sont dosés en routine mais un noyau dur de 4 marqueurs est partagé par tous les laboratoires: citrate, zinc (prostate), fructose (vésicules séminales) et α-1,4 glucosidase (épididyme). L’usage de méthodes marquées CE-IVD a concerné entre 0 et 75% de l’ensemble des dosages. Tous les laboratoires ont rendu des résultats de zinc dans les limites acceptables définies, 75% pour le citrate et α-1,4 glucosidase et 67% pour le fructose. En combinant toutes les données recueillies, nous avons construit un score qui a permis de classer le potentiel d’accréditation des marqueurs séminaux: l’accréditation du citrate, fructose et zinc ne devrait pas poser problème, l’ α-1,4 glucosidase présente une faiblesse analytique mais la phosphatase prostatique, la L-carnitine libre et la glycérophosphocholine ne sont pas accréditables en l’état actuel.

**Conclusion:**

Nous avons organisé un programme d’EEQ de biochimie séminale pour aider les laboratoires français à répondre à l’exigence d’accréditation totale en 2020. Ce programme est. perfectible mais il a permis de révéler les forces et faiblesses de ce paysage analytique. Des méthodes sont disponibles pour une exploration biochimique efficiente de la prostate et des vésicules séminales mais celle des épididymes est apparue particulièrement fragile. Ce problème andrologique doit être pris en compte par les autorités sanitaires et les sociétés savantes concernées.

## Background

Seminal plasma (SP) is a sperm-free fluid usually obtained in vitro after liquefaction and centrifugation of the ejaculated semen. It combines secretions from several glands of the male genital tract and constitutes over 90% of semen volume. Several biochemical components of the seminal plasma have been identified and quantified through specific assays since the pioneering works of Mann [1] and Lundquist [2]. They have been promptly proposed for the study of the pathophysiology of the male accessory genital glands [3, 4]. In the two last versions of its manual for the examination of human semen, WHO proposed some biochemical assays to assess the secretory capacity of these glands [5, 6]. For instance, zinc (Zn) and citric acid assays can explore the prostate; fructose is helpful for seminal vesicles while L-free carnitine, alpha-glucosidase and glycerophosphocholine (GPC) can assess epididymis presence and/or function.

In the last decades, the contribution of laboratory tests in the clinical decision-making has tremendously increased. Thus, their correct use and performance imply a multidisciplinary responsibility involving clinicians, laboratory medicine specialists, manufacturers and healthcare regulatory bodies [7]. To face this responsibility, an international effort for laboratory accreditation appeared in the late 2000’s and has spread since then [8]. Participation to relevant proficiency testing or external quality assessment (EQA) schemes is an essential requirement for accreditation. EQA programs, which were initially introduced as an educational tool, have evolved in scope and sophistication [9]. In standard conditions, the EQA organizing body sends a set of samples to participating laboratories for measurement of one or more biomarkers as they routinely do for clinical samples. Results are then returned to the EQA organizer which evaluates and compares them to the expected results and prepares for each participating laboratory a report that includes individual reported results, the method used, the assigned or target values for each biomarker and an evaluation of analytical performance of each method. It is now recognized that EQA programs that use commutable samples, i.e. which behaves as clinical samples during the measurement procedures, are particularly valuable and EQA providers are encouraged to publish such information [9]. In France, the law imposes that each laboratory, private or public, has to be fully accredited according to the ISO 15189 standard by 2020. In the fifth edition of its manual, the WHO underlined the need for a controlling for quality in the andrology laboratory by internal quality control and, when possible, by external quality control [6]. However, this injunction involves only fundamental parameters of sperm concentration, morphology and motility but not seminal biomarkers. To fill this gap and to help the French laboratories to face the legal requirements, we have organized an EQA program for seminal biochemical assays since 2014. The aim of this article is to provide an overview of the French laboratory testing provision for seminal biochemistry, focusing on assays analytical performance and the extent to which the requirements of ISO 15189 are met.

## Materials and methods

### EQA organizer

CTCB (Centre Toulousain pour le Contrôle de Qualité en Biologie clinique; www.ctcb.com) is a non-profit and independent association founded by clinical biochemists in 1973 in accordance with the French Law of 1901. The main goal of CTCB is to provide EQA schemes to medical laboratories and training programs to their staff. It manages 133 EQA schemes in clinical chemistry, microbiology, haematology, immunology, pharmacology and reproductive biology, each of them being directed by an expert of the field with the support of a scientific committee. CTCB is ISO/CEI 17043 accredited and collaborates with a wide EQA organizers network through the French federation FAEEQ (www.faeeq.fr) and the European organization EQALM (www.eqalm.org).

### EQA material

Specimens for EQA are derived from a pool of human SP made at the Biochemistry and Hormonology Laboratory of Toulouse University Hospital according to the following procedure: after routine assays for seminal biomarkers, 100 consecutive anonymized patients’ samples were stored at − 20 °C. Samples was residues of seminal plasmas obtained from patients attending the infertility clinic according to laboratory procedures and classical WHO recommendations (by masturbating, after sexual abstinence of 3 to 5 days,…) [6] and assayed for biomarkers. One week before the survey, the samples were thawed and pooled in an ice-chilled Erlen-Meyer. After 5 min of mixing, the pool was centrifuged during 10 min at 2000 g and 4 °C. Afterwards, the supernatant was harvested and aliquoted in 0.5 mL vials and stored at − 20 °C with no preservatives. A new pool is prepared for each survey. Preliminary test proved pools stability at − 20 °C for at least 2 years.

### Survey, procedures and data

Each participating laboratory was assigned an anonymous code number and received with the sample a form to fill out and to send back by e-mail or fax. The sample, a vial of 0.5 mL of human SP is shipped at − 20 °C. After receipt, laboratories processed their specimen as a patient sample and used their routine methods to assess the concentration of their own seminal biomarkers panel. Each survey proposes to assess citric acid or citrate and zinc for the prostate, fructose for seminal vesicles, alpha-glucosidase, free L-carnitine and GPC for epidydimis. For each biomarker, the requested data are: method, concentration and units. The results of seven surveys, from 2014 to 2019 were reported here. Survey of 2014 was a pilot operation that gave an overview on biomarkers panel and global analytical performances. That of 2018 included two specimen distributions. Since 2016, a clinical case about male infertility with 3 to 4 multiple choice questions was added to the survey and laboratories responses for this case were awaited in the same form that their analytical results.

### Target, imprecision and assessment of laboratories performance

Since the number of responses for each seminal biomarker was comprised between 3 and 7, the standard statistical plan of CTCB based on the NF ISO 13528 was not applicable. Thus, the consensus target value for each biomarker was defined as the all-laboratory median. The imprecision was calculated as the coefficient of dispersion (CD):

1 $$ CD=\frac{MAD}{median\times 100} $$

Where MAD is the median absolute deviation calculated as follow:

2 $$ MAD= median\ \left(\left|{x}_i\right.\left.- median\ \left({x}_i\right)\right|\right) $$

The CD is a robust measure of the variability of a univariate sample of quantitative data and can be used as an alternative of the coefficient of variation in case of nonnormal distribution in biological applications [10]. The use of z-score for estimating assays bias was not possible because of the low number of participants. Thus, we set the analytical performance specifications according to the model 3 of the Consensus Statement from the 1st Strategic Conference of the European Federation of Clinical Chemistry and Laboratory Medicine (or Milan Consensus) which is based on the state-of-the-art [11]. Since no data were available in the literature, we analyzed the pilot scheme of 2014 in order to discuss and to set the first allowable limits of performance (ALP) for biochemical markers of SP. Laboratories results were then reviewed and scored according to a procedure described in supplementary data file (Supplementary data S1).

## Results

### Number of laboratories and biomarkers panel

Between 2014 and 2019, a total of 42 laboratories, public or private, participated to the surveys with a median of 6 laboratories by survey (range, 5 to 7) and released a total of 173 results (median of 4 with a range of 2 to 6 results by laboratory). Amongst the proposed panel of seminal biochemical markers, only four could be evaluated (Table 1). No statistical analyses were possible for the other biomarkers [prostatic acid phosphatase (PAP), free L-carnitine, GPC, see Supplementary Table S1] because of the very low number of responses. For the prostate, citrate were assayed by a median of 5.5 laboratories (range, 4 to 7) and zinc by a median 3.5 ones (range, 1 to 5). For the seminal vesicles, fructose was assayed by a median of 6 laboratories (range, 5 to 7) and for epididymis, only α-1,4 glucosidase was frequently quantified (median of 5.5 with a range of 4 to 6). Thus, the core set of seminal assays was: citrate, zinc, fructose and α-1,4 glucosidase *i.e* at least one marker by annex gland. Noteworthy, two laboratories are able to analyze routinely 6 biomarkers amongst the following: citrate, zinc, prostatic acid phosphatase, fructose, free L-carnitine, α-1,4 glucosidase and GPC.

**Table 1:** Main results of 6 years and 7 surveys for the seminal biomarkers EQA program

Surveys	2014	2015	2016	2017	2018-A	2018-B	2019	Median
**Citrate**								
Median (*IQR*), mmol/L	27.60 (*5.27*)	24.00 (*2.96*)	26.60 (*0.87*)	29.1 (*0.1*)	27.59 (*4.42*)	28 (*4.06*)	28.80 (*2.3*)	**27.6**
CD, %	11.05	5.54	1.77	0.34	10	4.64	4.34	**4.64**
Number of laboratories	6	4	5	5	6	7	6	**6**
Consistent results^a^ (*%*)	3 (*50*)	3 (*75*)	5 (*100*)	4(*80*)	3 (*50*)	4 (*57*)	5 (*83*)	**75**^**b**^
**Zinc**								
Median (*IQR*), mmol/L	2.02 (*0.14*)	NA	1.70 (*0.14*)	2.31 (*0.30*)	2.20 (*0.57*)	2.00 (*0*)	2.25 (*0.26*)	**2.11**
CD, %	3.96	NA	7.65	10.82	11.8	0	8.44	**8.04**
Number of laboratories	5	1	3	3	4	5	5	**4**
Consistent results^a^ (*%*)	5 (*100*)	NA	3 (*100*)	3 (*100*)	3 (*75*)	5 (*100*)	4 (*80*)	**100**^**b**^
**Fructose**								
Median (*IQR),* mmol/L	14.43 (*3.2*)	17.76 (*3.23*)	14.65 (*1.00*)	16.20 (*0.80*)	15.00 (*1.29*)	16.00 (*2.45*)	16 (*1.78*)	**16**
CD, %	19.2	5.80	4.44	2.47	1.23	6.25	6.25	**5.8**
Number of laboratories	7	6	5	5	6	7	6	**6**
Consistent results^a^ (*%*)	2 (*29*)	2 (*33*)	5 (*100*)	5 (*100*)	4 (*67*)	5 (*71*)	4 (*67*)	**67** ^**b**^
**α-1,4 Glucosidase**								
Median (*IQR*), UI/L	20 (*2.2*)	17.65 (*4.4*)	17.60 (*2.96*)	21.85 (*2.60*)	19.16 (*3.21*)	20 (*0.75*)	18.75 (*4.5*)	**19.16**
CD, %	4	12.42	11.70	6.63	10.8	2.5	11.98	**10.8**
Number of laboratories	5	6	5	4	6	6	6	**6**
Consistent results^a^ (*%*)	4 (*67*)	4 (*67*)	3 (*60*)	3 (*75*)	5 (*83*)	5 (*83*)	5 (*83*)	**75** ^**b**^

^a^Consistent results are the overall compliant and acceptable results i.e. scored B-, A-, A+ or B+

^b^Calculated from % of consistent results

*CD* Coefficient of dispersion, *IQR* Interquartile range, *NA* Not available

### Methods used to quantify seminal biomarkers

These surveys provided us a unique vantage point on the methods that had been used to assay seminal biomarkers during the six last years (Supplementary Table 1). Citrate and fructose were always tested with enzymatic methods from industrial providers among which 60 and 58% were CE-IVD marked, respectively. Glucosidase was assayed with a mix of methods (1/3 in-house, 2/3 industrial) among which 32% were CE-IVD marked. For all surveys, no distinction was made between total or neutral α-1,4 glucosidase. Zinc was mainly tested by a spectroscopic-spectrometric approaches (2/3 of the assays) followed by colorimetric method. Only the later was CE-IVD marked (32%). Regarding other biomarkers, PAP and free L-carnitine were mainly tested with enzymatic CE-IVD marked methods (75 and 64% respectively) whereas GPC was only assayed with an in-house one.

### Laboratory performance: assays imprecision and results consistency

Assays imprecision of the main four biomarkers was assessed by calculating the median of the coefficients of dispersion (CD) (Table 1). Citrate CD was below 5%, those of zinc and citrate were between 5 and 10% and that of α-1,4 glucosidase was slightly below 10% (Table 1). Of interest, these figures are very close to the analytical specifications (ALP) initially set up for the surveys (Supplementary data 1). The highest proportion of consistent results was observed for zinc (100%), followed by those, equal, of citrate and α-1,4 glucosidase (75%) and finally by that of fructose (67%) (Table 1, last column).

### Target values for seminal biomarkers

For all surveys, the consensus target value for each biomarker was defined as the all-laboratory median. Since the specimen were obtained by pooling a high number of patients’ samples, we postulated that each biomarker ALM would be close to physiological concentrations. To confirm this hypothesis, we mined the literature and found five studies from 1991 to 2010 that reported seminal biomarkers concentrations for control patients i.e. fertile and/or normozoospermic men with normal ejaculate volume (Table 2). For the main four biomarkers (citrate, zinc, fructose and α-1,4 glucosidase), median, as well as minimum and maximum of ALM always fell within the reported interquartile ranges (Table 2).

**Table 2:** Comparison of biomarkers median values of seminal pools used in the EQA program with reference values reported in the literature [12–16]

	Results from surveys ^a^ Median (*Min-Max*)	1991 [12] Mean (*Q1-Q3*) ^b^	2000 [13] Median (*Q1-Q3*)	2002 [14] Median (*Q1-Q3*)	2004 [15] Mean (*Q1-Q3*)	2010 [16] Mean (*Q1-Q3*)
**Number of control patients** ^c^	NA	25	19	230	71	100
**Citrate** (mmol/L)	**27.6**	**27.8**	**28.6**	NR	NR	NR
	(*24.0–29.1*)	(*18.7–36.9*)	(*18.6–32.9*)			
**Zinc** (mmol/L)	**2.11**	**2.9**	NR	NR	NR	NR
	(*1.7–2.3*)	(*1.8–4.0*)				
**Fructose** (mmol/L)	**16**	**14.2**	**12.8**	NR	**13.9**	**14.6**
	(*14.4–17.8*)	(*10.0–18.4*)	(*10.0–18.2*)		(*9.4–17.6*)	(*11.2–18.0*)
**Total α-1,4 Glucosidase** (UI/L)	**19.2** (*17.6–21.9*)	**27.0** (*18.2–35.8*)	NR	**23.8** (*15.3–32.4*)	NR	NR
**Neutral α-1,4 Glucosidase** (UI/L)	NR	**19.5** (*12.1–26.9*)	**24.3** (*17.1–31.4*)	**20.2** (*12.3–28.1*)	NR	**14.1** (*6.9–21.3*)

^a^Extracted from Table 1

^b^Original data or derived from reported Mean and SD

^c^Fathers or normozoospermic patients

*NR* Not reported

### An attempt of methods scoring

The seven surveys provided pertinent data on how seminal biomarkers are actually assayed in France and their journey towards full accreditation. We have been trying to synthesize information through a scoring system based on the total number of assays performed, the median of CD, the proportion of consistent results and that of CE-IVD tests used (Supplementary Table 2). According to this empirical scoring, three biomarkers appear to be “good”: citrate, zinc and fructose. They are widely used with an overall satisfactory performance paving the way to accreditation. Surprisingly, α-1,4 glucosidase revealed a weakness: despite its wide use, there is still a high number of non-CE-IVD assays used by laboratories, a fact that may hamper the accreditation process. Finally, three biomarkers are “critical”: PAP, free L-carnitine and GPC mainly because they could not be evaluated in EQA surveys and will not be accredited in the current state.

## Discussion

In the present study, we analysed seven surveys of a French EQA program for seminal biomarkers that have been conducted over the last 6 years. We provided new data on the biomarkers panel offered by laboratories, the kind of assays used and their performance.

Nowadays, EQA programs represent a fundamental pillar of a laboratory’s quality management system. The first programs were introduced in laboratory medicine more than 70 years ago as educational tools to reveal that results for a same aliquot were different between laboratories [9]. Progressively, their scope was extended and some of them have reached a high level of design sophistication as those proposed by EQA providers that meet ISO 17043 requirements. Miller et al. proposed a classification of EQA programs into 6 categories according to how well they are able to evaluate performance [9]. Three characteristics underpin this evaluation capability: sample commutability, process for target assignment and inclusion or non-inclusion of replicate samples. Commutability of an EQA sample is a complex and much discussed property [9, 17]. A commutable sample has the same numeric relationship between measurement procedures than that of a representative set clinical samples. There are consensus procedures to validate this commutability but they are heavy and expensive to perform in current practice [9]. Since our EQA samples were obtained by pooling native and unmodified SP according to a well-described preparation, we reasonably assumed their commutability. For target values assignment (accuracy), we used the all-participants median value because there was no reference measurement procedures to trace results to. Finally, we did not include replicate samples that would permit to calculate individual intra-laboratory reproducibility. According to these characteristics, our EQA program belongs to the 4th category of Miller’s classification. For performance assessment of each biomarker assay, we set the limits around the target (ALP) values according to literature data and our own knowledge of seminal biochemistry. We will see below that those chosen ALP (S1) were fairly accurate. This state-of-art based approach fits to the model 3 of the Consensus Statement from the 1st Strategic Conference of the European Federation of Clinical Chemistry and Laboratory Medicine (or Milan Consensus) [11]. The 10 primary purposes of an EQA scheme have been recently recalled by Badrick et al. [17] amongst which the present EQA program fulfilled six (Supplementary Table 3). This overall examination highlights some strengths of the scheme (sample commutability, satisfying regulatory requirements) as well as some opportunities of improvement (moving to clinical and statistical-based performance assessment instead of state-of-art [18], assessing intra-lab reproducibility). However, since seminal biochemistry is an “analytical niche” involving a limited number of laboratories, it is unlikely that reference measurement procedures will be developed. This issue prevents our EQA to evaluate efficiently both accuracy and assays standardization.

A small number of laboratories (median of 6 by survey) have been regularly involved in the EQA programme, indicating that they routinely execute seminal plasma explorations and their willingness to accredit them. By way of comparison, around 90 laboratories routinely perform spermograms in France [19]. Thus, we could estimate two ratios: for 11.2 million French inhabitants, there is one laboratory performing biochemical assays versus 15 performing spermograms. Further studies are needed to determine whether this analytical offer matches the demand and to compare these ratios to those of other countries. Regarding the panel of biomarkers, we identified that a total of seven are routinely carried out: three for the prostate (citrate, zinc, prostatic acid phosphatase), one for the seminal vesicles (fructose) and three for epididymis (free L-carnitine, α-1,4 glucosidase and GPC). Interestingly, these biomarkers are those reported by the last WHO manual [6]. However only four assays are shared by all laboratories and could be evaluated in this EQA program: citrate, zinc, fructose, and α-1,4 glucosidase. Thus, each annex gland is explored by at least one biomarker. The EQA program allowed us to have also an overview on the used methods and their performances. There is a mix of in-house assays and industrial kits and their proportions are likely to vary from a biomarker to another. Citrate, PAP, Fructose and free-L Carnitine are mainly assayed with CE-IVD methods. For α-1,4 glucosidase, most of reported assays are in-house but a CE-IVD kit is available. For GPC, there is only an in-house method. The distinction between the two categories is of importance because the use of CE-IVD methods facilitates the accreditation process, standardization and methods traceability that are the gold standards of laboratory medicine. The case of zinc is particular since the most widely used methods are spectroscopy-based. Although they are not CE-IVD marked, they are recognized as efficient and valid than colorimetric methods for assessing zinc levels in human fluids [20]. Thus, citrate, PAP, fructose, α-1,4 glucosidase and free L-carnitine, for which CE-IVD assays are available can theoretically be carried out under ISO 15189 accreditation. In fact, according to the gathered data on the field, this may concern only citrate, fructose and zinc by extension. This unexpectedly provides a first clue on the fragility of the biochemical exploration of epididymis in France.

Regarding overall analytical performances, the median CD were below 10% for citrate, zinc and fructose and slightly above for α-1,4 glucosidase. Of interest, the reported values were very close to those of ALP initially expected to set laboratories performance (Supplementary data S1). Our EQA allows evaluating methods performance around the normal ranges as confirmed by Table 2 that reports seminal biomarkers concentrations for control patients i.e. fertile and/or normozoospermic men with normal ejaculate volume. It confirms that mixing a high number of seminal plasma samples provides physiological concentrations of biomarkers. Of interest, we noticed that reported interquartile ranges for total and neutral α-1,4 glucosidase were highly overerlapping. This point calls into question the value of distinguishing between total and neutral form of the enzyme in the clinical setting. In order to improve the EQA, samples with pathological levels, i.e. below thresholds proposed by the WHO manual [6], should be assayed in future surveys.

Finally, we undertook to synthesize all gathered data and to build a scoring system (Supplementary Table 2). We aimed to understand which biomarkers support laboratories efforts to meet ISO 15189 requirements and which do not. We reasoned that a “good” seminal biomarker: (i) is assayed by a high number of laboratories allowing a robust inter-laboratory comparisons; (ii) there are several CE-IVD methods with a good inter-method agreement; (ii) most laboratories provide consistent results indicating good skills and a mastery of methods. This reasoning is the rational of our scoring system. It appears that methods for citrate, zinc and fructose pave the way for meeting ISO 15189 requirements. It is less certain for α-1,4 glucosidase because it is more frequently analyzed with in-house methods that may extend its validation process. It seems completely out of the question for PAP, free L-carnitine and GPC that could not be evaluated in our EQA survey. Thus, one can consider the impact of these findings on andrological decision-making in France. Prostate and seminal vesicles are biochemically explored with a good level of confidence. Conversely, the situation is still far from satisfactory for epidydimis as we noted it above. To overcome this issue, it would be pertinent that each laboratory performs simultaneous assays of α-1,4 glucosidase and free-L carnitine with available CE-IVD methods.

## Conclusion

We organized an EQA program for seminal biomarkers to help French laboratories to face their legal requirement to be fully accredited by 2020. This program could be improved still further but gave us an oversight on the analytical landscape and revealed its strengths and weaknesses. Effective methods to assay citrate, zinc and fructose are available and permit confident exploration of prostate and seminal vesicles. However, there is a low number of involved laboratories and biochemical exploration of epididymis appeared unexpectedly fragile. This is an andrological issue and it would be advisable to solve it by dedicated recommendations from French health authorities and/or reproductive health societies.

## Supplementary information

**Additional file 1.** Setting analytical performance specifications for seminal biomarkers according to the Milan consensus model 3.

**Additional file 2: Table S1.** Overview of the analytical methods used to assay seminal biomarkers for the surveys.

**Additional file 3: Table S2.** Scoring of seminal biomarkers according to EQA results. **Table S3.** Primary purposes of an EQA scheme.

## Data Availability

Na

## Abbreviations

ALP: Allowable limits of performance
CD: Coefficient of Dispersion
CE-IVD: European CE Marking for In Vitro Diagnostic
EQA: External Quality Assessment
GPC: Glycerophosphocholine
IQR: Interquartile Range
ISO: International Organization for Standardization
PAP: Prostatic Acid Phosphatase
SP: Seminal Plasma
WHO: World Health Organization
Zn: Zinc
