# Supplementary material for: External quality assessment program for biochemical assays of human seminal plasma: a French 6-years experience
Source: Basic Clin Androl. 2020 Nov 17;30:18. doi: 10.1186/s12610-020-00116-2 (PMC7670731; doi:10.1186/s12610-020-00116-2)
Supplement: Supplementary file 1 — Additional file 1. Setting analytical performance specifications for seminal biomarkers according to the Milan consensus model 3. [file 12610_2020_116_MOESM1_ESM.docx]

Supplementary data S1

**Setting analytical performance specifications for seminal biomarkers according to the Milan consensus by the model 3 [11].**

By analysing the results from the pilot survey and discussion with several experts in the field, the first allowable limits of performance (ALP) were set for four biomarkers as following: ± 10% for α-1,4 glucosidase, ± 5% for citrate, ± 5% for fructose and ± 8% for zinc.

The result of a laboratory for a biomarker is considered to be compliant if it lies in the range : median ± 1 ALP ; acceptable if it lies in the range : median ± 2 ALP. Between 2 and 3 ALP, the laboratory should reconsider its results and beyond ± 3 ALP, the result is not compliant. In order to help the laboratories, their results are scored with a letter (A, B, C, D) and a sign (+ or -) which respectively indicate the analytical performance and the position relative to the median according to the following table below.

**
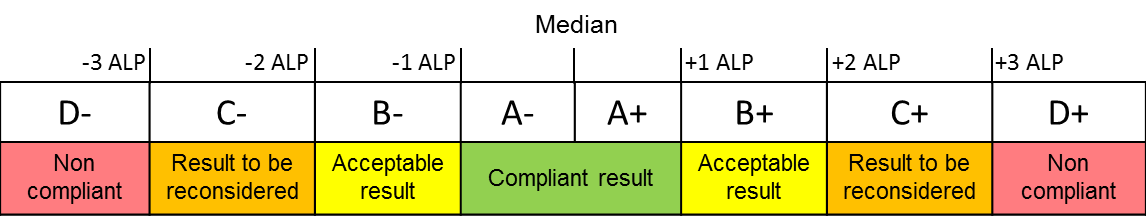
**
