# Supplementary material for: External quality assessment program for biochemical assays of human seminal plasma: a French 6-years experience
Source: Basic Clin Androl. 2020 Nov 17;30:18. doi: 10.1186/s12610-020-00116-2 (PMC7670731; doi:10.1186/s12610-020-00116-2)
Supplement: Supplementary file 2 — Additional file 2: Table S1. Overview of the analytical methods used to assay seminal biomarkers for the surveys. [file 12610_2020_116_MOESM2_ESM.docx]

**SUPPLEMENTARY TABLE 1:** Overview of the analytical methods used to assay seminal biomarkers for the surveys.

| **Analytes** | **Providers/Suppliers** | **Type of Assay** | **Number**  **of responses** | **Rounds** | ***CE-IVD^a^* marking** |
| --- | --- | --- | --- | --- | --- |
| CITRATE | BIOSENTEC | Enzymatic | 14 | 2015, 2016, 2017, 2018A-B, 2019 | Yes |
|  | FERTIPRO | Enzymatic | 10 | 2014, 2015, 2017, 2018A-B, 2019 | Yes |
|  | LIBIOS | Enzymatic | 1 | 2014 | No |
|  | R-BIOPHARM | Enzymatic | 8 | 2015, 2016, 2017, 2018A-B, 2019 | No |
|  | ROCHE Boehringer Mannheim | Enzymatic | 6 | 2014, 2015, 2016, 2017, 2019 | No |
|  | Unknown | - | 1 | 2014 | - |
|  | **TOTAL** |  | **40** |  | **24** (*60%*) |
| FRUCTOSE | BIOSENTEC | Enzymatic | 16 | 2015, 2016, 2017, 2018A-B, 2019 | Yes |
|  | FERTIPRO | Enzymatic | 9 | 2016, 2017, 2018A-B, 2019 | Yes |
|  | LIBIOS | Enzymatic | 2 | 2014, 2015 | No |
|  | LTA Srl | Enzymatic | 1 | 2015 | ? |
|  | R-BIOPHARM | Enzymatic | 10 | 2014, 2016, 2017, 2018A-B, 2019 | No |
|  | ROCHE Boehringer Mannheim | Enzymatic | 3 | 2015, 2017, 2019 | No |
|  | Unknown | - | 2 | 2014 | - |
|  | **TOTAL** |  | **43** |  | **25** (*58%*) |
| α-1,4 GLUCOSIDASE | FERTIPRO | Colorimetric | 12 | 2014, 2015, 2016, 2017, 2018A-B 2019 | Yes |
|  | In house assay | Colorimetric | 15 | 2014, 2015, 2016, 2017, 2018A-B 2019 | No |
|  | ROCHE Boehringer Mannheim | Colorimetric | 10 | 2014, 2015, 2016, 2017, 2018A-B, 2019 | No |
|  | Unknown | - | 1 | 2014 | - |
|  | **TOTAL** |  | **38** |  | **12** (*32%*) |
| ZINC | Spectroscopic method | AAS^b^ | 11 | 2014, 2015, 2016, 2017, 2018A-B, 2019 | No |
|  | Spectrometric method | ICP-MS^c^ | 6 | 2016, 2017, 2018A-B, 2019 | No |
|  | WAKO | Colorimetric | 10 | 2014, 2015, 2016, 2018A-B, 2019 | Yes |
|  | Unknown | - | 2 | 2014, 2017 | - |
|  | **TOTAL** |  | **29** |  | **10** (*34%*) |
| Free L-CARNITINE | In house assay | Enzymatic | 4 | 2014, 2017, 2018B | No |
|  | BIOSENTEC | Enzymatic | 7 | 2015, 2016, 2017, 2018A-B, 2019 | Yes |
|  | **TOTAL** |  | **11** |  | **7** (*64%*) |
| GPC^d^ | In house assay | Enzymatic | 8 | 2014, 2015, 2016, 2017, 2018A-B, 2019 | No |
|  | **TOTAL** |  | **8** |  | **0** (*0%*) |
| PAP^e^ | In house assay | Colorimetric | 1 | 2014 | No |
|  | BIOLABO  ROCHE Diagnostics GmBH | Colorimetric  Colorimetric | 2  1 | 2015, 2016  2019 | Yes  Yes |
|  | TOTAL |  | **4** |  | **3** (*75%*) |

^a^CE-In vitro Diagnostic, ^b^Atomic absorption spectroscopy, ^c^Inductively coupled plasma – mass spectrometry , ^d^Glycerophosphocholine, ^e^Prostatic acid phosphatase.
