# Supplementary material for: External quality assessment program for biochemical assays of human seminal plasma: a French 6-years experience
Source: Basic Clin Androl. 2020 Nov 17;30:18. doi: 10.1186/s12610-020-00116-2 (PMC7670731; doi:10.1186/s12610-020-00116-2)
Supplement: Supplementary file 3 — Additional file 3: Table S2. Scoring of seminal biomarkers according to EQA results. Table S3. Primary purposes of an EQA scheme. [file 12610_2020_116_MOESM3_ESM.docx]

**SUPPLEMENTARY TABLE 2:** Scoring of seminal biomarkers according to EQA results.

| **Biomarker** | Number  of assays | *Score 1** | CE-IVD | *Score 2* | CD | *Score 3* | Consistent results | *Score 4* | Total Score |
| --- | --- | --- | --- | --- | --- | --- | --- | --- | --- |
| Citrate | 40 | *6* | 60% | *5* | 4.64% | *7* | 75% | *6* | **24** |
| Zinc | 29 | *4* | 34% | *3* | 8.04% | *5* | 100% | *7* | **19** |
| PAP | 4 | *1* | 75% | *7* | ND | *0* | ND | *0* | **8** |
| Fructose | 43 | *7* | 58% | *4* | 5.8% | *6* | 67% | *5* | **22** |
| α-1, 4 glucosidase | 38 | *5* | 32% | *2* | 10.8% | *4* | 75% | *6* | **17** |
| Free L-carnitine | 11 | *3* | 64% | *6* | ND | *0* | ND | *0* | **9** |
| Glycerophosphocholine | 8 | *2* | 0% | *1* | ND | *0* | ND | *0* | **8** |

*Scoring method: Biomarkers were scored according to their rank for each variable obtained throughout the surveys (columns). The best marker for a variable was scored 7, the second 6…etc. *Ex-aequo* biomarkers were scored equally. A non-ranked biomarker was scored 0. A global score was obtained by adding scores 1 to 4. The maximum score is 28. Three tertiles are defined: above 19, the biomarker is qualified “**good**”, between 10 and 18, “**weak**” and below 10, “**critical**”.

**SUPPLEMENTARY TABLE 3:** Primary purposes of an EQA scheme (according to [17]).

| 1 | Characterises test bias and imprecision across methods |
| --- | --- |
| 2 | Correlates specific method variables with bias and imprecision |
| 3 | Identifies interfering substances and quantify their effects across multiples methods |
| **4** | **Provides clinical laboratories with reliable information for replacing unsatisfactory methods*** |
| **5** | **Identifies clinical laboratories that are at risk for poor performance*** |
| **6** | **Satisfies accreditation and regulatory requirements*** |
| 7 | Assessment of method robustness to clinically relevant interference |
| **8** | **Assessment of individual laboratory performance*** |
| **9** | **Communication with participating laboratories*** |
| **10** | **Audit of wider aspects of analytical performance and educational activities*** |

**Specific purposes that are fulfilled by the EQA for biochemical markers of seminal plasma are highlighted in bold.*
